# Factors affecting reorganisation of memory encoding networks in temporal lobe epilepsy

**DOI:** 10.1016/j.eplepsyres.2014.11.001

**Published:** 2015-02

**Authors:** M.K. Sidhu, J. Stretton, G.P. Winston, M. Symms, P.J. Thompson, M.J. Koepp, J.S. Duncan

**Affiliations:** a Department of Clinical and Experimental Epilepsy, UCL Institute of Neurology, Queen Square, London WC1 N 3BG, UK; b Epilepsy Society MRI Unit, Chesham Lane, Chalfont St. Peter SL9 0RJ, UK

**Keywords:** Temporal lobe epilepsy, Episodic memory, Functional MRI, Seizure frequency, Duration

## Abstract

**Aims:**

In temporal lobe epilepsy (TLE) due to hippocampal sclerosis reorganisation in the memory encoding network has been consistently described. Distinct areas of reorganisation have been shown to be efficient when associated with successful subsequent memory formation or inefficient when not associated with successful subsequent memory. We investigated the effect of clinical parameters that modulate memory functions: age at onset of epilepsy, epilepsy duration and seizure frequency in a large cohort of patients.

**Methods:**

We studied 53 patients with unilateral TLE and hippocampal sclerosis (29 left). All participants performed a functional magnetic resonance imaging memory encoding paradigm of faces and words. A continuous regression analysis was used to investigate the effects of age at onset of epilepsy, epilepsy duration and seizure frequency on the activation patterns in the memory encoding network.

**Results:**

Earlier age at onset of epilepsy was associated with left posterior hippocampus activations that were involved in successful subsequent memory formation in left hippocampal sclerosis patients. No association of age at onset of epilepsy was seen with face encoding in right hippocampal sclerosis patients. In both left hippocampal sclerosis patients during word encoding and right hippocampal sclerosis patients during face encoding, shorter duration of epilepsy and lower seizure frequency were associated with medial temporal lobe activations that were involved in successful memory formation. Longer epilepsy duration and higher seizure frequency were associated with contralateral extra-temporal activations that were not associated with successful memory formation.

**Conclusion:**

Age at onset of epilepsy influenced verbal memory encoding in patients with TLE due to hippocampal sclerosis in the speech-dominant hemisphere. Shorter duration of epilepsy and lower seizure frequency were associated with less disruption of the efficient memory encoding network whilst longer duration and higher seizure frequency were associated with greater, inefficient, extra-temporal reorganisation.

## Highlights

• Earlier age of onset of epilepsy was associated with reorganisation of memory encoding function to the posterior temporal lobe, including the posterior hippocampus.
• Shorter duration of epilepsy and lower seizure frequency were associated with activations in the anterior medial temporal lobes.
• Longer duration of epilepsy and greater seizure frequency were associated with extra-temporal activations.
• These findings contribute to preoperative risk counselling for epilepsy surgery.

## Introduction

Temporal lobe epilepsy (TLE) is associated with impaired episodic memory. In healthy individuals functional imaging has shown material specific activation patterns, left hemispheric activation for verbal memory encoding and right for visual (Golby et al., 2002). In TLE, reorganisation of the memory encoding network to the contralateral medial temporal lobe has been consistently described (Bonelli et al., 2010; Figueiredo et al., 2008; Powell et al., 2007; Richardson et al., 2003; Sidhu et al., 2013).

Memory in TLE has been related to several clinical factors including duration of epilepsy (Cheung et al., 2006), age at onset and seizure frequency (Hermann et al., 2002; Kaaden and Helmstaedter, 2009). Age at onset and duration of epilepsy have also been shown to be predictive of memory outcome after anterior temporal lobe resection (Baxendale et al., 2006; Baxendale, 2008).

We recently showed areas of increased temporal and extra-temporal activations during memory encoding in patients with left and right unilateral hippocampal sclerosis (HS) compared to healthy controls, a process that has been consistently described as functional reorganisation (Sidhu et al., 2013). By performing an event-related subsequent memory analysis and correlations with out of scanner neuropsychometry measures we further classified these encoding activations as being associated with successful subsequent memory formation, and encoding activations not associated with successful subsequent memory formation (Sidhu et al., 2013).fMRI studies have shown that memory reorganisation within medial temporal lobe structures are influenced by age at onset of epilepsy, epilepsy duration and seizure frequency. Cheung et al. (2006) showed that longer duration of epilepsy was associated with reduced medial temporal lobe activation whilst greater reorganisation to the contralateral medial temporal lobe has been associated with a lower seizure frequency (Figueiredo et al., 2008). In a non-material specific complex scene encoding task, earlier age at onset of epilepsy was associated with greater asymmetry of medial temporal lobe activations, conferred by predominant contralateral activations (Mechanic-Hamilton et al., 2009). To date, factors influencing extra-temporal memory reorganisation have not been investigated.

To summarise the results of our 2013 study, we showed an ipsilateral reduction in network activation during both verbal and visual encoding. Left HS patients showed greater contralesional temporal and extra-temporal activations compared to controls across both verbal and visual encoding whilst right HS patients showed bilateral increases. In both LHS and RHS patients encoding activations within the contralateral medial temporal lobe during word and face encoding were associated with successful subsequent memory formation. Extra-temporally, encoding activations within the left orbitofrontal and anterior cingulate cortex in LHS patients during word encoding and left insula activation in RHS patients during face encoding were associated with successful subsequent memory formation.

In this study we aimed to investigate the effect of age at onset of epilepsy, duration of epilepsy and the effect of seizure burden on both temporal and extra-temporal memory reorganisation. We hypothesised that:

(1) Patients with an earlier age at onset, longer duration of epilepsy and greater seizure frequency would have greater alteration of the memory encoding network with predominantly ipsilesional temporal and extra-temporal reduction in activations and more extensive reorganisation contralateral to the lesion.
(2) Patients with a shorter duration of epilepsy, later age at onset and lower seizure frequency would have less disruption of the memory encoding network with greater activations in brain areas shown to be associated with subsequent memory formation (Sidhu et al., 2013).

## Materials and methods

### Subjects

We studied 53 patients with medically refractory TLE (29 left: median age 40 years (range 19–54), 24 right: median age 42.5, range 21–56) due to unilateral HS having pre-surgical evaluation at the National Hospital for Neurology and Neurosurgery, London. All patients had undergone structural MRI at 3T, including quantification of hippocampal volumes and T2 relaxation times confirming unilateral HS with normal contralateral medial temporal structures (Table 1). Prolonged inter-ictal EEG and ictal video-EEG telemetry confirmed ipsilateral seizure onset in all patients. All patients were on antiepileptic medication and spoke fluent English. These patients were in our previous study, with the addition of a further five left hippocampal sclerosis (LHS) and four right hippocampal sclerosis (RHS) patients (Sidhu et al., 2013). 26 healthy native English speaking controls, median age 37 years (range 19–58) with no neurological or psychiatric history were also studied. This study was approved by the National Hospital for Neurology and Neurosurgery and the Institute of Neurology Joint Research Ethics Committee. Written informed consent was obtained from all participants. Handedness and language dominance were determined using a standardised questionnaire (Oldfield, 1971) and language fMRI (Powell et al., 2006) respectively using language lateralisation within an inferior and middle frontal gyrus mask (Bonelli et al., 2010; Sidhu et al., 2013).

Seizure frequency was calculated as the number of complex partial seizures per month. There was no significant difference between patient groups in age, age at onset of epilepsy, epilepsy duration, seizure frequency, hippocampal volumes, number of AEDs or language lateralisation (Independent sample two-tailed *t*-test, *p* > 0.05, Table 1).

### Memory testing

All patients underwent standardised clinical memory testing using the BIRT Memory and Information Processing Battery (BMIPB) verbal learning (VL) and design learning (DL) subtests (Coughlan et al., 2007) previously shown to be sensitive to MTL dysfunction (Baxendale et al., 2006). For verbal learning 15 words are presented for learning over five trials. A distractor list is then presented and delayed recall of the original list is tested. In the visual learning task a nine element design is presented for learning over five trials with delayed recall assessed after a distracting design. Four scores were generated: verbal learning, verbal delayed recall, design learning and design delayed recall.

### Statistical analysis

Statistical analyses were performed using PASW Statistics 18.0 (IBM, Armonk, USA). Correlations between age, age at onset of epilepsy, duration, hippocampal volume, number of antiepileptic drugs (AED), frequency of complex partial seizures (CPS) per month and language lateralisation were performed with the verbal and visual memory test scores described above.

### Magnetic resonance data acquisition

Studies were performed using a 3 T General Electric Excite HDx MRI scanner: maximum gradient strength of 40 mT m^−1^, slew rate 150 T m^−1^ s^−1^. For the fMRI, gradient-echo echo planar images were acquired, providing blood oxygen level dependent contrast (BOLD). Each volume comprised 36 contiguous oblique axial slices, slice thickness 2.5 mm (0.3 mm gap), field of view 24 cm, matrix 96 × 96 interpolated to 128 × 128 during image reconstruction, reconstructed voxel size 1.875 × 1.875 × 2.5 mm, resolution 2.5, SENSE factor 2.5, Echo time 25 ms, TR 2.75 s. The field of view was positioned to cover the temporal and frontal lobes with the slices aligned with the long axis of the hippocampus on the sagittal view.

### Memory encoding paradigm

Faces and words were visually presented to patients during a single scanning session (Sidhu et al., 2013).

Black and white photographs of non-famous faces unfamiliar to the subjects and single concrete nouns were presented on an MR compatible screen viewed via a mirror. Each item was presented for 3 s in 60 s blocks. We used a different inter-stimulus interval (3 s) to our TR of 2.75 s to introduce jitter and facilitate random sampling.

Each block consisted of 10 faces and 10 words followed by 15 s cross hair fixation and the initial presentations of either faces or words were counterbalanced.

We presented a total of 10 blocks (100 faces and 100 words). Participants were explicitly instructed to memorise items for subsequent out of scanner recall. A deep encoding task (Craik, 2002) that involved a subjective decision on whether each stimulus was pleasant or unpleasant was performed.

Forty minutes later face and word recognition were tested separately in an out-of-scanner recognition task. In each task, subjects were shown the same 100 items randomly intermixed with an additional 50 novel faces/words. A button box was used to indicate if items were remembered, familiar or novel and in the scanner stimuli were subsequently sorted into these three groupings, remembered, familiar and forgotten.

### Data analysis

Analysis was performed using SPM8 (http://www.fil.ion.ucl.ac.uk/spm/). The imaging time series was realigned, normalised into standard anatomical space (using a scanner specific template created from 30 healthy controls, 15 patients with left HS and 15 patients with right HS using the high resolution whole brain EPI) and smoothed with a Gaussian kernel of 8 mm full-width at half maximum.

Using a blocked design, regressors of interest were formed by creating two box-car functions for faces and words convolved with the canonical HRF. Contrasts were generated for both ‘faces’ and ‘words’ corresponding to the main effect of material-specific encoding at the first level. A random effects analysis was performed at the second level. Subjects were divided into three groups: controls, LHS and RHS. A one-sample *t*-test was performed to examine the group effect of each contrast. An analysis of variance (ANOVA) was performed to quantitatively assess statistically different brain activations between all three groups.

Efficiency of reorganised networks was previously assessed using an event-related analysis where trial specific delta functions were convolved with the canonical HRF and its temporal derivative (Sidhu et al., 2013). Six regressors of interest for each of the event types, words remembered (WR), words familiar (WFam), words forgotten (WF), faces remembered (FR), faces familiar (FFam) and faces forgotten (FF) were created. Movement parameters were included as confounds. Contrast images were created for each subject for verbal subsequent memory (defined by (WR) − (WFam + WF)) and visual subsequent memory (defined by (FR) − (FFam + FF)). These images were used for the second-level analysis.

At the second level, one-sample t-tests were used to examine the group effect of each subsequent memory contrast in each group. Verbal and visual subsequent memory activations from the event-related analyses were correlated with verbal (VL) and design learning (DL) scores respectively in controls, LHS and RHS patients. Correlations were explored using memory scores as a continuous regressor in an analysis of covariance (ANCOVA) (Sidhu et al., 2013).

## Factors affecting memory reorganisation

The effect of age at onset of epilepsy, epilepsy duration and seizure frequency on word and face memory encoding networks was explored separately in LHS and RHS patients. These parameters were entered as continuous regressors in an ANCOVA against the word and face whole brain blocked design encoding activations in each patient group.

As age at onset was highly correlated to duration of epilepsy (Pearson correlation coefficient 0.75, *p* < 0.001), the ANCOVA was performed with either age at onset or duration used as a covariate to investigate the effect of these clinical parameters in turn on face and word encoding networks in LHS and RHS patients. The number of complex partial seizures per month at the time of the scan was used as a measure of current seizure frequency. As there was no correlation between age at onset or duration of epilepsy and seizure frequency (Pearson correlation < 0.5), neither of these were used as an additional covariate in this analysis.

Results of these regression analyses are presented in this manuscript as a follow on to the findings of the blocked and event-related analyses that were recently published (Sidhu et al., 2013). All results for main effects of the blocked design activations for word and face encoding in controls, LHS and RHS patients were reported corrected for multiple comparisons, family wise error (FWE), *p* < 0.05 (Sidhu et al., 2013). Regression analyses were reported at *p* < 0.005 uncorrected. Medial temporal lobe activations were reported corrected for multiple comparisons, FWE *p* < 0.05 using a small volume correction within a 12 mm diameter sphere (Bonelli et al., 2010; Sidhu et al., 2013).

## Results

### Neuropsychological memory test performance

There was no significant difference between the patient groups on the verbal memory measures (Independent sample *t*-test, *p* > 0.05) but LHS patients performed significantly better than RHS patients in the design learning and delayed design recall tasks (Independent sample two-tailed *t*-test *p* < 0.01), (Table 1).

In LHS patients, neither verbal list learning nor delayed verbal recall correlated with age at onset, duration of epilepsy, seizure frequency, hippocampal volumes or language lateralisation (*p* > 0.05).

In RHS patients, shorter duration of epilepsy was associated with better design learning (Pearson correlation 0.57, *p* = 0.007). Neither design learning nor delayed design recall correlated with age at onset, language lateralisation, seizure frequency or hippocampal volumes (*p* > 0.05).

### fMRI results: Regression analyses

#### Age at onset

##### Word encoding

**Fig. 1:**
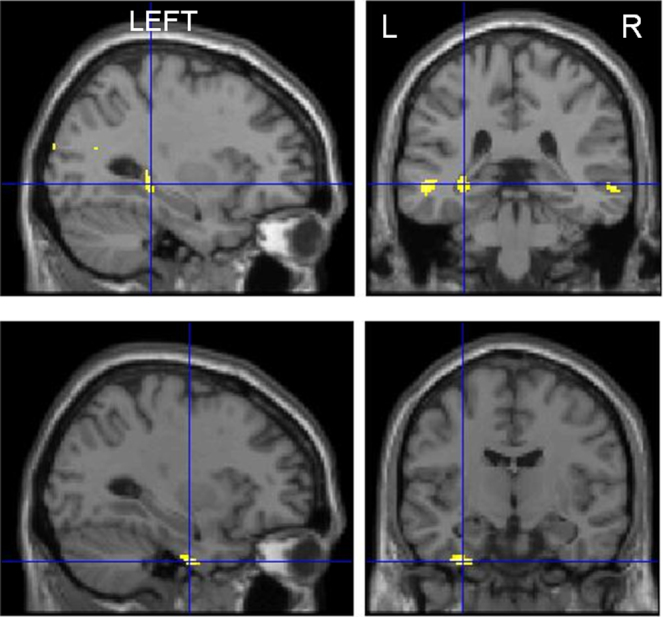
Correlation of word encoding with age at onset. Upper panel: left posterior hippocampal and bilateral posterior medial temporal lobe activations associated with an earlier age at onset of epilepsy. Lower panel: left anterior fusiform gyrus activations associated with older age at onset of epilepsy.

In LHS patients, earlier age at onset was associated with significant activations within the posterior left hippocampus, middle occipital gyrus, post-central gyrus and bilateral posterior middle temporal gyri. Older age at onset was associated with left anterior fusiform gyrus activations (Fig. 1, Suppl. Table 1). No significant correlation of age at onset was seen in RHS patients during word encoding.

##### Face encoding

Neither LHS nor RHS patients showed a significant correlation between face encoding and age at onset of epilepsy.

#### Duration of epilepsy

##### Word encoding

**Fig. 2:**
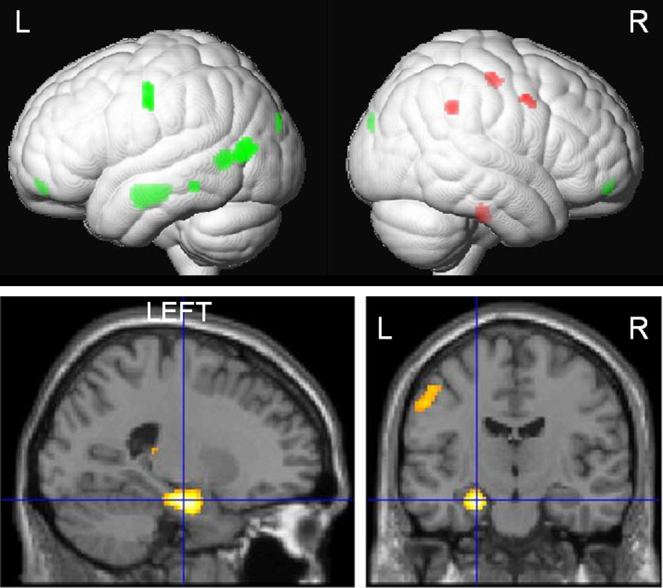
Correlation of word encoding with duration of epilepsy. Upper panel: render image showing predominantly left hemispheric activations (green) associated with a shorter duration and right hemispheric activations (red) associated with longer duration of epilepsy. Lower panel: sagittal and axial image showing left medial temporal lobe activations associated with a shorter duration of epilepsy. (For interpretation of the references to color in this figure legend, the reader is referred to the web version of this article.)

In LHS patients, longer duration correlated significantly with right hemispheric activations within the pre-central gyrus, middle frontal gyrus and supramarginal gyrus. Shorter duration correlated predominantly with left hemispheric activations in hippocampus, mid occipital and temporal gyri (MTG), postcentral gyrus, medial orbitofrontal gyrus (OFG) and inferior frontal gyri (IFG). Significant right parahippocampal gyrus (PHG) correlation was also seen. No significant correlation was seen in RHS patients (Fig. 2, Suppl. Table 2).

##### Face encoding

In LHS patients, shorter duration of epilepsy correlated significantly with right amygdala and left hippocampus, OFG and superior temporal gyrus (STG) activations. Longer duration of epilepsy correlated with activations within the right supramarginal gyrus, pre-central gyrus and left inferior parietal lobule.

**Fig. 3:**
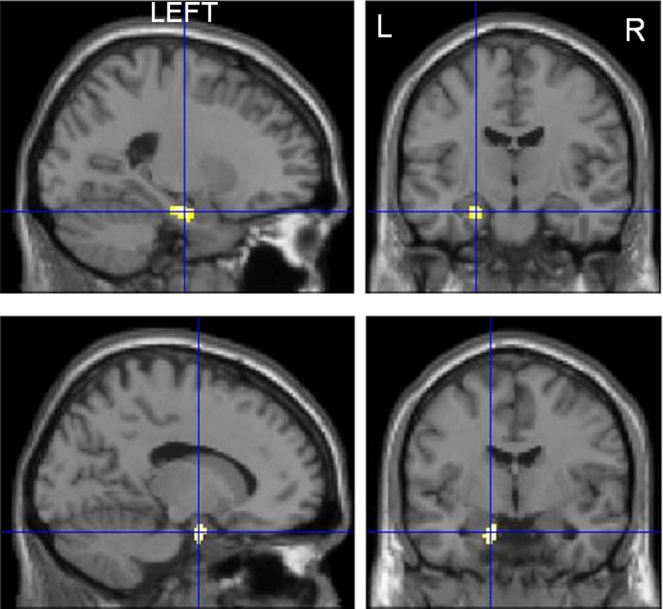
Correlation of face encoding with shorter duration of epilepsy. Left medial temporal lobe activations associated with a shorter duration of epilepsy in LHS patients (upper panel) and RHS patients (lower panel) during face encoding.

In RHS patients, shorter duration correlated with left PHG and hippocampal activations whilst longer duration correlated with left post-central gyrus activation (Fig. 3, Suppl. Table 2).

#### Seizure frequency

##### Word Encoding

In LHS patients lower seizure frequency was associated with activations within the left IFG, right STG, PHG, rolandic operculum and bilateral hippocampal activations. Higher seizure frequency correlated with right OFG activation.

**Fig. 4:**
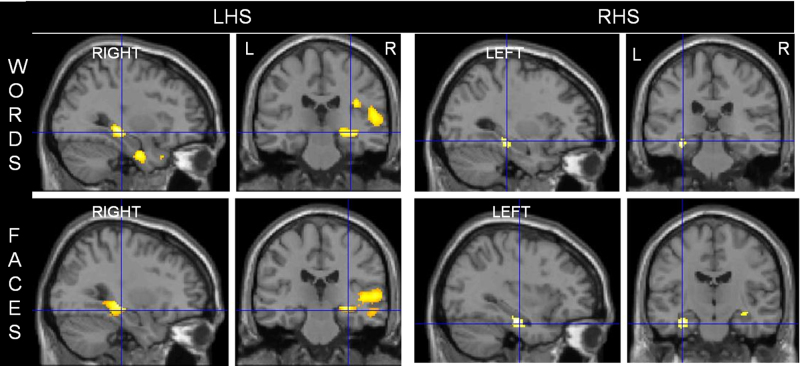
Correlation of lower seizure frequency with word (upper panel) and face encoding (lower panel) in LHS and RHS patients. Predominantly right medial and lateral temporal lobe activations associated with a lower seizure frequency in LHS patients during word encoding and face encoding. Conversely, predominantly left medial temporal lobe activations associated with lower seizure frequency in RHS patients during word and face encoding.

In RHS patients, lower seizure frequency was associated with left MTL activation within the PHG and hippocampus whilst higher frequency was associated with activation within the left rolandic operculum (Fig. 4, Suppl. Table 3).

##### Face encoding

In LHS patients, lower seizure frequency correlated with activations within the right hippocampus, STG, MTG, left amygdala and bilateral IFG. Higher seizure frequency correlated with activations within the left paracentral lobule.

In RHS patients, lower seizure frequency correlated significantly with activations within the left PHG and HC and right amygdala and PHG whilst a higher seizure frequency correlated with activations within the left post-central gyrus and inferior parietal lobule (Fig. 4, Suppl. Table 3).

## Discussion

### Summary of findings

Earlier age at onset of epilepsy influenced the word encoding network in LHS patients. Patients with earlier age of onset showed left posterior hippocampus, bilateral posterior middle temporal gyrus and occipital activations.

Duration of epilepsy and seizure frequency significantly influenced both verbal and visual memory encoding. In LHS patients, shorter duration of epilepsy was associated with bilateral MTL activations during word and face encoding whilst in RHS patients, shorter duration was associated with left MTL activation during face encoding. LHS patients showed particularly left extra-temporal activations correlating with shorter duration for both word and face encoding whilst longer duration in both LHS and RHS patients associated with contralesional extra-temporal reorganisations.

In LHS patients, contralateral MTL activations were associated with lower seizure frequency for both word and face encoding. In RHS patients, bilateral MTL activations during face encoding and left MTL activations during word encoding were associated with a lower seizure frequency. Higher seizure frequency was associated with greater extra-temporal activations.

### Age at onset of epilepsy

Previously, we showed that activations in the left posterior hippocampus and posterior middle temporal gyri bilaterally were associated with successful verbal memory formation in LHS patients (Sidhu et al., 2013). In this study we showed that this reorganisation was influenced by an earlier age at onset of epilepsy. Early onset of epilepsy appears to compromise the normal functioning of the anterior left hippocampus. Age at onset was not seen to influence word encoding in RHS patients or face encoding in either patient group.

Post-natal brain development continues into adolescence with brain myelination occurring in a posterior to anterior fashion so frontal lobe myelination occurs last. In TLE patients, early age at onset of epilepsy affected white matter maturation with a lag in the frontal and parietal but not the occipital lobes (Hermann et al., 2010).

The hemispheric asymmetry of brain maturation suggests that the left hemisphere matures later than the right. Regional cerebral blood flow at rest was maximal in the right hemisphere at 1 year and in the left hemisphere at age 3, in keeping with the cadence of skill acquisition in children namely visuo-spatial (right dominant) skills followed by language acquisition (left dominant) (Chiron et al., 1997). Disruption of this maturation process by early onset epilepsy may therefore preferentially influence ‘dominant’ functions including verbal memory. Another possible explanation for the modulation of the verbal encoding network only in LHS patients is that structural and morphological changes described in early onset epilepsy may be greater in left TLE than in right TLE patients (Kemmotsu et al., 2011; Riederer et al., 2008).

This hemispheric and anterior-posterior maturation asymmetry renders the left hemisphere and frontal lobes more vulnerable to early insults such as that incurred from early seizures which may explain why there appeared to be a selective effect of earlier age at onset of epilepsy in LHS patients.

### Duration of epilepsy

In TLE patients, Cheung et al. (2006) showed reduced MTL activations associated with longer duration of epilepsy in a scene encoding task where controls showed bilateral MTL activations. This task was not material specific therefore contralateral activation due to reorganisation could not be assessed. Using a material specific paradigm, we showed that in a left lateralised task of word encoding in LHS patients a longer duration of epilepsy was associated with less MTL activation. Conversely, longer duration correlated with contralateral extra-temporal activations that we previously showed to be unrelated to successful subsequent memory formation (Sidhu et al., 2013). Shorter duration correlated with left extra-temporal hemispheric activation including the left OFG during verbal encoding. We previously showed that left OFG activations in LHS patients during verbal encoding were associated with successful subsequent memory formation (Sidhu et al., 2013).

In RHS patients during face encoding, shorter duration of epilepsy correlated with left MTL activation whilst longer duration correlated with left post-central gyrus activations that were not associated with subsequent memory formation in our previous study (Sidhu et al., 2013). This implies that longer duration of epilepsy is associated with greater disruption of the MTL functions in individuals with LHS and RHS with more engagement of neocortex that is not efficient (Sidhu et al., 2013). In RHS patients, this corroborates the finding that better visual memory correlated with shorter duration of epilepsy. In pharmacoresistant epilepsy, anterior temporal lobe resection has up to a 80% chance of resulting in seizure remission (de Tisi et al., 2011) and early surgical intervention has been shown to prevent permanent disability (Engel, 2008). Our findings of greater disruption of the encoding networks with longer duration of epilepsy support early consideration of surgical treatment.

### Seizure frequency

Pathological studies have suggested that repeated complex partial seizures might cause neuronal injury (Dam, 1980) and negatively impact on cognition in TLE (Hermann et al., 2002).

We did not find that current seizure frequency correlated with memory test performance or hippocampal volume but we found significant correlations between seizure frequency and fMRI activations during verbal and visual encoding. Lower complex partial seizure frequency correlated with anterior MTL activations in both LHS and RHS patients during face and word encoding. In LHS patients lower seizure frequency correlated with involvement of the right MTL; an activation previously shown to be associated with subsequent memory formation (Sidhu et al., 2013). Fewer seizures also correlated with left IFG and left HC activations that have been shown to be associated with successful verbal memory formation in controls (Hongkeun, 2011).

In RHS patients lower complex partial seizure frequency was associated with greater activation within the left PHG at word encoding and bilateral PHG on face encoding, activations that we showed to be associated with successful subsequent memory in right HS patients in our previous study (Sidhu et al., 2013).

In both LHS and RHS patients during word and face encoding higher seizure frequency was associated with extra-temporal activations that were not associated with successful memory formation (Sidhu et al., 2013). Our findings concur with a study of 10 RHS patients, in which a lower seizure frequency correlated with MTL activation that was relevant to subsequent recognition performance during visual encoding (Figueiredo et al., 2008).

The overall inference is that, in TLE with HS, more frequent seizures are associated with greater disruption of normal encoding networks and the recruitment of other cerebral areas did not compensate for this.

### Strengths and limitations

We included a large homogenous cohort of HS patients and applied robust analysis methods and used conservative statistical thresholds that allow inferences to be made about HS patients as a population. As age at onset and duration are highly correlated clinical variables we performed all correlation analyses controlling for each of these factors in turn.

We estimated current complex partial seizure frequency as the best measure of seizure frequency in our patients but this may not be a true reflection of total lifetime seizure burden. Gender was fairly balanced in the LTLE group (16 female and 13 male) but not in the RTLE group (5 female and 19 male). This discrepancy was not accounted for in our analysis. We investigated only patients with TLE and HS therefore generalisability to other forms of TLE remains to be investigated.

We report our findings as a group inference. Further research using objective parameters would be necessary to be able to apply these findings to an individual patient.

### Neurobiological and clinical implications

We showed that age at onset of epilepsy, epilepsy duration and seizure frequency significantly affected memory encoding networks in TLE patients. Early age at onset was associated with successful memory reorganisation to the left posterior medial and bilateral lateral temporal lobes. Shorter duration of epilepsy and fewer complex partial seizures were associated with MTL and neocortical activations predictive of successful subsequent memory formation whilst longer epilepsy duration and greater burden of seizures were associated with ‘inefficient’ neocortical activations.

Anterior temporal lobe resection for refractory TLE has up to an 80% chance of inducing seizure remission for years at a time (de Tisi et al., 2011). An important consideration, however, is the memory deficits that may ensue. The patterns of organisation and reorganisation of memory encoding networks visualised using fMRI have been shown to be an important predictor of memory decline following surgery (Bonelli et al., 2010; Powell et al., 2008; Richardson et al., 2004). Bonelli et al. (2010) showed that preoperative reorganisation of both verbal and visual memory to the posterior hippocampus was associated with a lower risk of memory decline post-operatively and activation in the ‘to-be resected’ anterior hippocampus was predictive of memory decline post-operatively. We show that age at onset, duration of epilepsy and seizure frequency, significantly influence memory reorganisation and these factors may contribute to the prediction of memory outcome after surgery.

## Conclusion

Earlier age of onset of epilepsy influences verbal memory reorganisation in left HS patients, being associated with left posterior hippocampal and temporal activations. Duration of epilepsy and seizure frequency significantly influenced memory encoding networks in both LHS and RHS patients during verbal and visual encoding. Shorter duration and lower seizure frequency were associated with memory activations in brain regions that were involved in successful memory formation, with longer duration and higher seizure frequency associated with extra-temporal memory reorganisation to brain regions that did not contribute to successful memory formation. Investigating the pre-operative memory encoding network and understanding factors that influence memory reorganisation has important implications when considering the possible effects of epilepsy surgery on memory.

## Figures and Tables

**Table 1:** Demographic details and results of standard memory tests in patients shown as mean (SD). LI (lateralisation index), HV (hippocampal volume), L15 (verbal list learning), L6 (delayed verbal recall), D15 (design learning), D6 (delayed visual recall), ^*^ LHS > RHS Independent sample *t*-test, *p* < 0.01, AED (anti epileptic drug).

	Age (yrs)	Age at onset (yrs)	Durat ion [yrs)	fMRI Lang uage LI	L15 (/75)	L6 (/15)	D15 (/45)	D6/9	RHV cm^3^	LHV cm^3^	CPS/mth	AED
LHS	40 (7.5)	14.6 (10.9)	23.8 (14.4)	−0.69 (0.3)	43.5 (10.4)	7.9 (3.1)	34^*^ (7.4)	7.2^*^ (1.7)	2.8 (0.4)	1.8 (0.4)	8.4 (10.8)	2 (1)
RHS	42.5 (14.5)	13.2 (10.3)	29.1 (16.2)	−0.64 (0.4)	43.3 (9.8)	9.3 (3)	28.3 (7.8)	5.5 (3)	1.9 (0.4)	2.7 (0.3)	5.7 (6)	2 (1)
