# Supplementary material for: Factors affecting reorganisation of memory encoding networks in temporal lobe epilepsy
Source: Epilepsy Res. 2015 Feb;110:1–9. doi: 10.1016/j.eplepsyres.2014.11.001 (PMC4315807; doi:10.1016/j.eplepsyres.2014.11.001)
Supplement: Supplementary file 1 [file mmc1.docx]

| **WORD ENCODING LHS Patients: Earlier age at onset** | | | | | | | | |
| --- | --- | --- | --- | --- | --- | --- | --- | --- |
|  | **Left Hemisphere** | | | | **Right Hemisphere** | | | |
| **Region** | **Coordinate** | | **P value** | **Zscore** | **Region** | **Coordinate** | **Pvalue** | **Zscore** |
| Mid Occipital G | -50 -78 10 | | 0.000 | 3.40 | Inf Temporal G | 44 -52 -8 | 0.001 | 3.21 |
| Hippocampus | -26 -20 -18 | | 0.002* | 2.89 | Mid Temporal G | 60 -36 -6 | 0.002 | 2.91 |
| Mid Temporal G | -54 -34 -6 | | 0.003 | 2.80 |  |  |  |  |
| Post. Hippocampus | -30 -36 0 | | 0.036* | 2.78 |  |  |  |  |
| Post- Central G | -56 -12 38 | | 0.003 | 2.77 |  |  |  |  |
| **Left HS Patients: Later age at onset** | | | | | | | | |
|  | **Left Hemisphere** | | | | **Right Hemisphere** | | | |
| **Region** | **Coordinate** | **P value** | | **Zscore** | **Region** | **Coordinate** | **Pvalue** | **Zscore** |
| Fusiform G | -30 -10 -40 | 0.000 | | 3.72 |  |  |  |  |

Supplementary Table 1: Coordinates, p-values and z-scores of whole brain activations associated with earlier and later age at onset of epilepsy in LHS patients. * Family wise error corrections p<0.05 using a small volume correction within a sphere of 12mm in diameter for medial temporal lobe activations activations. Mid (middle), Inf (inferior), Post (posterior) G (gyrus).

| **WORD ENCODING** | | | | | | | |
| --- | --- | --- | --- | --- | --- | --- | --- |
| **LHS Patients: Shorter duration of epilepsy** | | | | | | | |
|  | **Left Hemisphere** | | | **Right Hemisphere** | | | |
| **Region** | **Coordinate** | **P value** | **Zscore** | **Region** | **Coordinate** | **Pvalue** | **Zscore** |
| Hippocampus | -22 -14 -20 | 0.000* | 4.51 | Parahippocampal | 18 0 -20 | 0.048* | 2.54 |
| Mid Occipital G | -42 -62 4 | 0.000 | 3.46 |  |  |  |  |
| Mid Temporal G | -50 -72 6 | 0.001 | 2.99 |  |  |  |  |
| Post-central G | -54 -12 40 | 0.001 | 3.18 |  |  |  |  |
| Med Orbito-frontal | -4 58 -14 | 0.001 | 3.01 |  |  |  |  |
| Inf Frontal G | -36 16 12 | 0.003 | 2.74 |  |  |  |  |
| **LHS Patients: Longer Duration of epilepsy** | | | | | | | |
| ` |  |  |  | Pre-central G | 24 -18 58 | 0.000 | 3.63 |
|  |  |  |  | Mid Frontal G | 32 4 44 | 0.003 | 2.78 |
|  |  |  |  | Supramarginal G | 40 -44 36 | 0.004 | 2.63 |

| **FACE ENCODING LHS Patients: Shorter duration of epilepsy** | | | | | | | |
| --- | --- | --- | --- | --- | --- | --- | --- |
|  | **Left Hemisphere** | | | **Right Hemisphere** | | | |
| **Region** | **Coordinate** | **P value** | **Zscore** | **Region** | **Coordinate** | **Pvalue** | **Zscore** |
| Hippocampus | -24 -14 -20 | 0.000 | 3.52 | Amygdala | 26 -2 -28 | 0.036* | 2.61 |
| Orbito-Frontal G | -30 56 -8 | 0.005 | 2.58 |  |  |  |  |
| Sup Temporal G | -38 22 -32 | 0.003 | 2.72 |  |  |  |  |
| **LHS Patients: Longer duration of epilepsy** | | | | | | | |
|  | **Left Hemisphere** | | | **Right Hemisphere** | | | |
| **Region** | **Coordinate** | **P value** | **Zscore** | **Region** | **Coordinate** | **Pvalue** | **Zscore** |
| Inf. Parietal L | -58 -30 50 | 0.000 | 3.54 | Supramarginal G | 44 -42 32 | 0.000 | 3.58 |
|  | -24 -10 44 | 0.001 | 3.28 | Pre-central G | 24 -18 58 | 0.001 | 3.01 |
| **FACE ENCODING RHS Patients: Shorter duration of epilepsy** | | | | | | | |
|  | **Left Hemisphere** | | | **Right Hemisphere** | | | |
| **Region** | **Coordinate** | **P value** | **Zscore** | **Region** | **Coordinate** | **Pvalue** | **Zscore** |
| Parahippocampal G | -14 -2 -24 | 0.026* | 2.74 |  |  |  |  |
| **RHS Patients: Longer duration of epilepsy** | | | | | | | |
| Post-central G | -42 -36 54 | 0.005 | 2.55 |  |  |  |  |

| **WORD ENCODING LHS Patients: lower CPS frequency** | | | | | | | |
| --- | --- | --- | --- | --- | --- | --- | --- |
|  | **Left Hemisphere** | | | **Right Hemisphere** | | | |
| **Region** | **Coordinate** | **P value** | **Zscore** | **Region** | **Coordinate** | **Pvalue** | **Zscore** |
| Hippocampus | -20 -20 -14 | 0.027* | 2.77 | Hippocampus | 36 -28 -6 | 0.001* | 4.08 |
| Inf Frontal G | -42 12 28 | 0.003 | 2.73 | Parahippocampal G | 30 -6 -32 | 0.001* | 3.76 |
|  |  |  |  | Sup Temporal G | 56 -22 8 | 0.001 | 3.11 |
|  |  |  |  | Rolandic operculum | 38 -26 24 | 0.001 |  |
| **LHS Patients: higher CPS frequency** | | | | | | | |
|  |  |  |  | OFC | 18 60 -10 | 0.001 | 3.13 |
| **RHS Patients: higher CPS frequency** | | | | | | | |
| Rolandic operculum | -42 -34 22 | 0.004 | 2.64 |  |  |  |  |
| **RHS Patients: lower CPS frequency** | | | | | | | |
| PHG/hippocampus | -32 -28 -14 | 0.026* | 2.93 |  |  |  |  |

Supplementary Table 2: Coordinates, p-values and z-scores of whole brain activations associated with duration of epilepsy during word encoding in LHS patients and face encoding in LHS and RHS patients. There was no correlation of epilepsy duration and word encoding in RHS patients. * Family wise error corrections p<0.05 using a small volume correction within a sphere of 12mm in diameter for medial temporal lobe activations. Sup (Superior), Mid (Middle), Inf (Inferior), Med (Medial), G (gyrus), L (lobule).

| **FACE ENCODING LHS Patients: lower CPS frequency** | | | | | | | |
| --- | --- | --- | --- | --- | --- | --- | --- |
|  | **Left Hemisphere** | | | **Right Hemisphere** | | | |
| **Region** | **Coordinate** | **P value** | **Zscore** | **Region** | **Coordinate** | **Pvalue** | **Zscore** |
| Inf Temporal G | -46 -50 -10 | 0.000 | 3.52 | Mid Temporal G | 60 -48 -6 | 0.000 | 4.77 |
| Inf Frontal G | -42 12 28 | 0.003 | 2.79 | Hippocampus/PHG | 28 -28 -6 | 0.000* | 4.28 |
| Amygdala |  | 0.038* | 2.59 | Sup Temporal G | 58 -24 4 | 0.000 | 3.78 |
|  |  |  |  | Inf Frontal G | 48 18 24 | 0.003 | 2.73 |
| **LHS Patients: higher CPS frequency** | | | | | | | |
| Paracentral lobule (/WM) | -12 -32 58 | 0.000 | 3.33 |  |  |  |  |
| **RHS Patients: lower CPS frequency** | | | | | | | |
| PHG/ Hippocampus | -32 -18 -20 | 0.034* | 2.65 | Amygdala/PHG | 36 0 -24 | 0.026* | 2.76 |
|  |  |  |  | Hipopocampus | 24 -12 -18 | 0.021* | 2.55 |
| **RHS Patients: higher CPS frequency** | | | | | | | |
| Post-central G | -48 -12 32 | 0.001 | 3.14 |  |  |  |  |
| Inf Parietal L | -46 -32 24 | 0.002 | 2.95 |  |  |  |  |

Supplementary Table 3: Coordinates, p-values and z-scores of whole brain activations associated with seizure frequency during face and word encoding in LHS and RHS patients.* Family wise error corrections p<0.05 using a small volume correction within a sphere of 12mm in diameter for medial temporal lobe activations. CPS (complex partial seizure), Sup (Superior), Inf (inferior), G (gyrus), PHG (parahippocampal gyrus), Mid (middle), L (lobule)
